# Reproducibility in patient‐specific IMRT QA

**DOI:** 10.1120/jacmp.v15i3.4741

**Published:** 2014-05-08

**Authors:** Elizabeth M. McKenzie, Peter A. Balter, Francesco C. Stingo, Jimmy Jones, David S. Followill, Stephen F. Kry

**Affiliations:** ^1^ The University of Texas Graduate School of Biomedical Sciences at Houston Houston TX; ^2^ Department of Radiation Oncology The Methodist Hospital Houston TX; ^3^ Department of Radiation Physics Radiological Physics Center, The University of Texas MD Anderson Cancer Center Houston TX USA

**Keywords:** reproducibility, IMRT, quality assurance, patient‐specific QA

## Abstract

The purpose of this study was to determine the reproducibility of patient‐specific, intensity‐modulated radiation therapy (IMRT) quality assurance (QA) results in a clinical setting. Six clinical patient plans were delivered to a variety of devices and analyses, including 1) radiographic film; 2) ion chamber; 3) 2D diode array delivered and analyzed in three different configurations (AP delivery with field‐by‐field analysis, AP delivery with composite analysis, and planned gantry angle delivery); 4) helical diode array; and 5) in‐house‐designed multiple ion chamber phantom. The six clinical plans were selected from a range of treatment sites and were of various levels of complexity. Of note, three of the plans had failed at least preliminary evaluation with our in‐house IMRT QA; the other three plans had passed QA. These plans were delivered three times sequentially without changing the setup, and then delivered two more times after breaking down and rebuilding the setup between each. This allowed for an investigation of reproducibility (in terms of dose, dose difference or percent of pixels passing gamma) of both the delivery and the physical setup. This study showed that the variability introduced from the setup was generally higher than the variability from redelivering the plan. Radiographic film showed the poorest reproducibility of the dosimeters investigated. In conclusion, the various IMRT QA systems demonstrated varying abilities to reproduce QA results consistently. All dosimetric devices demonstrated a reproducibility (coefficient of variation) of less than 4% in their QA results for all plans, with an average reproducibility of less than 2%. This work provides some quantification for the variability that may be seen for IMRT QA dosimeters.

PACS numbers: 87.55.Qr, 87.56.Fc

## I. INTRODUCTION

Intensity‐modulated radiation therapy (IMRT) has become ubiquitous in radiation therapy clinics. The increased complexity of IMRT plans necessitates a quality assurance (QA) approach that departs from the traditional hand calculation‐based verification. IMRT patient plans are routinely validated in the clinic by using direct measurement for each patient. To satisfy this need, a number of devices have been developed to measure doses from IMRT patient plans, which are then compared with the intended dose distribution calculated by the treatment planning system (TPS).

For the sake of convenience, several metrics have been adopted that allow for the sorting of plans as passing (the delivered dose distribution adequately reflects the intended dose distribution, as calculated by the TPS) or failing. Two of these metrics are percent difference and percent of pixels passing the gamma criterion.^(1)^ Percent difference is often used with point measurements, such as with an ion chamber, whereas gamma analysis is used for planar measurements, such as film or a diode array. The institution chooses a threshold value for these metrics to indicate whether the plan might or might not be suitable for delivery to a patient. However, the credibility of this sorting rests in part on the reproducibility of the sorting, which in turn rests on the reproducibility in the delivery of the plan and the dose measurements. A robust IMRT QA system, therefore, requires good reproducibility of the measured dose.

Previously published studies have explored the reproducibility of individual measurements. For example, Mancuso et al.^(2)^ investigated the reproducibility of the ion chamber, film, and 2D diode array measurements in patient‐specific IMRT QA. However, this work was based on the structure set geometries given in TG119,^(3)^ not actual clinical plans. Fraser et al.^(4)^ investigated the reproducibility of various ion chambers for IMRT QA. However, no prior study, to the authors' knowledge, has explored the reproducibility of IMRT QA results on clinical IMRT plans compared across a wide array of devices. This study, therefore, explores the variation in the measured dose and QA results for several IMRT QA devices subjected to repeated measurements and analysis, and also divides the reproducibility into that associated with the delivery process and the device setup.

## II. MATERIALS AND METHODS

### A. Plans

Six different step‐and‐shoot IMRT patient plans that had previously undergone patient‐specific IMRT QA were selected from the authors' institutional database. To select for varying degrees of complexity, three of the plans were chosen from a pool of plans that had previously failed our internal film and ion chamber‐based QA;^(5)^ the other three plans had previously passed the QA. The three failing plans included one from thoracic (THOR 1), one from head and neck (HN 1), and one from gynecological (GYN 1), while the three passing plans included two from thoracic (THOR 2 and THOR 3) and one from gastrointestinal (GI 1). All plans were calculated in Pinnacle^3^ version 9 software (Philips Medical Systems, Andover, MA), developed with the same TPS beam model, with a 3 mm dose grid, using exclusively 6 MV photons.

### B. D elivery methods

With the exception of the ArcCHECK (Sun Nuclear Corporation, Melbourne, FL), all plans were delivered on the same Varian 21 iX accelerator (Varian Medical Systems, Palo Alto, CA).

Because the ArcCHECK was housed in a different building, the same accelerator could not be used; plans were therefore delivered to this device with an equivalent, and dosimetrically matched, machine. For any given dosimeter, all measurements were taken within a single evening and accounted for the machine output.

Four commercial dosimeters and one in‐house‐designed dosimeter were used for this study: EDR2 radiographic film (Kodak Carestream, Rochester, NY), Wellhofer cc04 ion chamber (CNMC, Nashville, TN), MapCHECK2 2D diode array (Sun Nuclear Corporation), ArcCHECK helical diode array (Sun Nuclear Corporation), and an in‐house‐designed multiple ion chamber (MIC) phantom.

**Figure 1:**
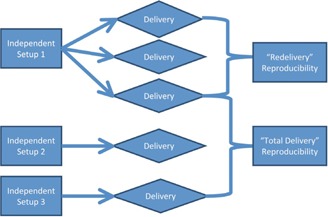
The workflow used to generate measurements for “total delivery” and “redelivery” reproducibility. This same workflow was performed on all six patient plans on all IMRT QA devices studied.

After initial setup, each plan was delivered three times to each dosimeter. The system was then perturbed, reset up, and reirradiated (for a fourth time), and then perturbed, reset up, and reirradiated again (for a fifth time). All measurements were set up by the same operator, using the in‐room lasers to guide setup. Ultimately, this yielded three irradiations delivered under a each patient plan on every device. The third irradiation from the “redelivery” measurements was counted as one of the independent setup measurements for “total delivery.” The “redelivery” measurements were performed to assess the reproducibility of the machine delivery, device readout, and the analysis associated with that QA dosimeter. The “total delivery” measurements incorporate both the variability seen in the “redelivery” measurements, as well as the variability introduced from the setup of the equipment. Figure 1 is a visual representation of this workflow.

The film could not be directly reirradiated as with the other dosimeters. While a new sheet of film had to be placed within the phantom for each irradiation, for the “redelivery” measurements the phantom was not moved on the table. The film was localized using pinpricks built into the phantom to ensure that each new film irradiation was analyzed in the same phantom setup geometry.

### C. Dosimeters used

The EDR2 radiographic film and cc04 ion chamber were placed in an I'm*RT* body phantom (IBA Dosimetry, Schwarzenbruck, Germany) with the film placed in the transverse plane. The phantom was shifted to allow the ion chamber to have an average dose of at least 70% of the maximum dose in the plan, and a standard deviation equal to or less than 1% of the mean value dose across the active volume of the ion chamber, in accordance with the practices performed at the authors' institution. The goal of this procedure is to place the ion chamber in a high‐dose, low‐gradient region. All plans were delivered with their original gantry angles.

Because radiographic film and an ion chamber make fundamentally different measurements with differing geometries, these two dosimeters were considered separately in assessing their reproducibility. For the film, a gamma analysis in OmniPro I'm*RT* (IBA Dosimetry) was done with a 10% low‐dose threshold and criteria of 3%/3 mm (global dose difference) for the reproducibility analysis. Before performing the gamma analysis, a region of interest (ROI) was selected to include only that region of film within the phantom, and the scanned film plane was aligned using pinpricks from the phantom. Also, the film was normalized to a user defined point to reasonably maximize the agreement between the film and TPS calculation.

The MapCHECK2 diode array was placed in a MapPHAN phantom to provide 5 cm of water‐equivalent buildup and 5 cm for backscatter. The device was then irradiated and analyzed in three different configurations. First, it was irradiated with gantry angles set to zero degrees for an AP beam delivery and a field‐by‐field gamma analysis was done. Second, it was irradiated with gantry angles set to zero degrees for an AP beam delivery and a composite field gamma analysis was done. Third, it was irradiated with the original planned gantry angles and composite field gamma analysis was done. For this third condition, per the manufacturer's instructions, if the majority of angles for any plan approached 90° or 270°, the array would have been placed sagittally. However, since this was not the case for any of the plans being investigated (none had the majority of its beams coming from a more liberal range of either 70°–100° or 250°–290°), all plans were delivered with the MapCHECK flat on the treatment couch. For all three conditions, the gamma analysis was done at 3%/3 mm (global dose difference) in the DoseLab Pro software (Mobius Medical Systems, Houston, TX), with an automatically selected ROI and using absolute dose (calibration fields were used to calibrate the MapCHECK for absolute dose mode; the results of these calibration fields were adjusted by the linear accelerator output, which was determined with an ion chamber immediately prior to calibration of the MapCHECK). The auto ROI algorithm created a rectangle containing all points receiving more than 30% of the maximum dose. The ROI is an expansion of this rectangle by 10% of its width and height. The measured plane was the reference distribution in the gamma analysis.

The ArcCHECK helical diode array was positioned on the couch, with shifts away from or toward the gantry, as was necessary, to avoid irradiation of the electronics and to center the treatment plan on the center diodes. Gamma analysis was performed with use of SNC patient software (Sun Nuclear Corporation) at 3%/3 mm (global dose difference), with an ROI that encompassed all of the diodes, using absolute dose mode, and using the measured plane as the reference distribution in the gamma analysis.

The in‐house designed MIC phantom consisted of five Exradin A1SL ion chambers (Standard Imaging Inc., Middleton, WI) set at three‐dimensionally independent points (different depths, heights, and lateral positions). The position of the ion chambers was optimized such that the number of ion chambers in a high‐dose, low‐gradient region was maximized. Each ion chamber was completely independent, having its own triaxial cable and electrometer. The measured dose from each ion chamber was used in the reproducibility analysis. The coefficient of variation was calculated for each of the five ion chambers and then averaged over each patient plan to arrive at a summary statistic.

### D. Methods of analysis

The data were collected in “redelivery” and “total delivery” conditions, as defined above. The standard deviation and coefficient of variation for each patient plan on each IMRT QA dosimeter for both types of reproducibility (”redelivery” and “total delivery”) was then determined. In this paper, the reproducibility is given in terms of the coefficient of variation (standard deviation divided by the mean). The “total delivery” measurement captures the variation introduced by the delivery/readout and the setup; these two components can be added in quadrature on the reasonable assumption that these errors are uncorrelated because they relate to independent processes (Eq. (1)).

(1) $$\sqrt{\sigma_{redeliver\mathrm{y m}easurements}^{2}+\sigma_{setup}^{2}}=\sigma_{tota\mathrm{l r}edeliver\mathrm{y m}easurements}$$

Therefore, reproducibility in the setup can be extracted from the “total delivery” measurement by removing the “redelivery” measurement component. This was done to determine how much variability was derived from the setup and how much from the delivery/readout.

Follow‐up analysis was also performed to explore the relationship between positioning guidelines for point dosimetry and the reproducibility in such measurements. Ion chambers are accepted as a trusted standard in the field of dosimetry;^(6)^ however, in patient‐specific IMRT QA, the ion chamber is often utilized outside of reference conditions.^(4)^ There is an assumption that minimizing the gradient across the ion chamber and placing the ion chamber in a high‐dose region is associated with a more reproducible measurement. A common method of determining whether the chamber is in an acceptable gradient is to analyze the standard deviation of the dose across the ion chamber volume as calculated by the TPS. By comparing the standard deviation in the dose across the ion chamber active volume with the measured standard deviation in repeated measurements, our study analyzed that assumed relationship. This was done by plotting the standard deviations of the dose across the ion chamber ROI as calculated in Pinnacle^3^ 9.0 versus the standard deviations found in the measurements. To also test the impact of dose gradients in the local environment around the ion chamber, similar analysis was done by looking at the standard deviation of the dose across the ion chamber ROI expanded (in 3 dimensions) by 1, 2, 3, 4, and 5 mm. The R statistical package (R Foundation for Statistical Computing, Vienna, Austria) was used to perform regression analysis for all ROIs. In addition to these tests of the effects of a dose gradient, the effects of dose level were also evaluated (whether the dose level for the ion chamber predicted reproducibility of the signal). A similar regression analysis comparing the standard deviation in the measurement to the percent of the plan maximum dose was performed to analyze this relationship.

## III. RESULTS

**Figure 2:**
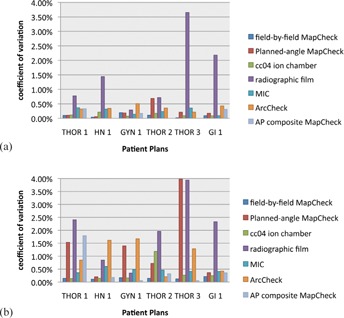
”Redelivery” (a) and “total delivery” (b) reproducibility for each dosimeter system, grouped by patient plan (including the multiple ion chamber (MIC) phantom). All methods showed a coefficient of variation of less than 4%, with film demonstrating the highest variability for both delivery/readout and “total delivery” reproducibility, with “total delivery” reproducibility including variation from both the delivery/readout and setup.

Table 1 is provided to show the average measurement values obtained for each QA device, before processing the data to analyze its reproducibility. These averages are taken across all six patient plans, and are stated separately for both the “redelivery” and “total delivery” measurements. Figure 2 shows the “redelivery” and “total delivery” reproducibility for each QA system measured for each patient plan; in these analyses, “total delivery” reproducibility included the overall uncertainty from setup, delivery, and readout of results. The coefficient of variation describes the variability in the absolute dose measurement (for the ion chamber readings) or the percent of pixels passing gamma (for the planar/array devices). It should be noted that only a 3%/3 mm gamma criterion was used in this study. While this was selected due to its common use, a choice of different criterion could possibly lead to a different reproducibility. A salient feature of these plots is the heterogeneity in a dosimeter's coefficient of variation across the various plans. Although plan “THOR 3” showed the overall highest coefficient of variation for both the “redelivery” and “total delivery,” no plan‐based statistical difference was found when an ANOVA was run (p=0.88). This indicates that plan‐dependent characteristics were not particularly important in terms of device reproducibility, at least for this varied sample of patient plans. Rather, reproducibility was determined more by the device, as detailed below.

**Table 1:** Device measurement averages taken across all six patient plans. The measurement used for the reproducibility analysis is given in parentheses (percent of pixels passing gamma for the planar devices, the nC charge reading for the cc04 ion chamber, and the dose (cGy) measured for the multiple ion chamber (MIC) phantom. The averages reported are for both the “redelivery” and “total delivery” measurements (three measurements each), which over the six patients translates to averages taken over 18 measurements each for “redelivery” and “total delivery”

*QA System (Format of Measurement)*	*Redelivery*	*Total Delivery*
AP field‐by‐field MapCHECK (% pixels passing gamma)	99.0	99.0
Planned‐angle MapCHECK (% pixels passing gamma)	96.4	95.7
cc04 ion chamber (charge in nC)	2.49	2.49
Radiographic film (% pixels passing gamma)	93.4	92.3
ArcCHECK (% pixels passing gamma)	93.1	93.3
AP composite MapCHECK (% pixels passing gamma)	98.6	98.3
MIC (dose in cGy)	167	167

Figure 2(a) shows the variety of “redelivery” reproducibility exhibited by the various devices due to machine fluctuations, dosimeter readout, and analysis. All devices, except film, demonstrated consistent QA results between repeated deliveries, as shown by the relatively small bars. For example, the AP composite MapCHECK had a “redelivery” coefficient of variation of 0% for three plans (the exact same percent of pixels passing were obtained for each delivery under the same setup). In contrast, the radiographic film generally had much higher variability in the measurements. Apart from film, all other QA systems showed less than 1% variation for “redelivery” reproducibility (Fig. 2(a)), indicating little variation in the delivery/readout portion of the QA.

The “total delivery” reproducibility (Fig. 2(b)) shows a greater spread in device performance. This relationship shows how the reproducibility of these devices may have similar consistency in the readout of their results, but their different setups can lead to QA system‐based differences in the constancy of QA results.

**Figure 3:**
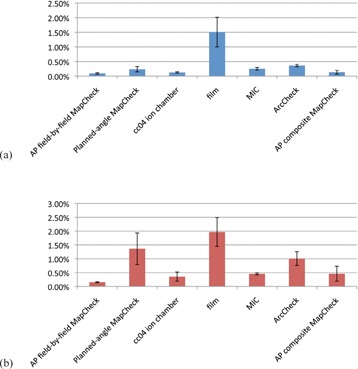
Patient‐averaged “redelivery” (a) and “total delivery” reproducibility (b) coefficients of variation (CV) for each device (including the multiple ion chamber (MIC) phantom). The errors bars are given as standard error. The film shows the highest variability of all the devices.

**Figure 4:**
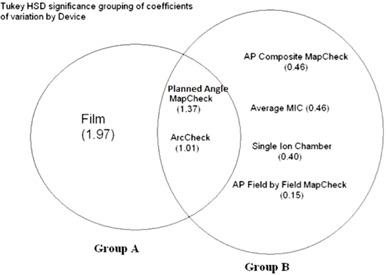
Venn diagram showing statistically significant grouping of dosimetric systems based on their “total delivery” reproducibility. The coefficient of variation is given in parentheses. The coefficient of variation of film was significantly different from the cc04 ion chamber, MapCHECK with AP beams formed into a composite plane, MapCHECK with AP beams analyzed in a field‐by‐field analysis, and the multiple ion chamber (MIC) phantom. The coefficient of variation of the ArcCHECK and the MapCHECK with the original rotational gantry angles were not significantly different from either group.

To examine specifically the devices' performance, the coefficient of variation for each patient plan was averaged across each device. Figure 3(a) displays the “redelivery” average coefficient of variations per device, with the standard error shown as error bars. Figure 3(b) similarly shows the “total delivery” average coefficient of variation with standard error bars. Figure 3(a) shows that the radiographic film demonstrates a clearly higher variability in QA results compared with the other dosimeter systems. An ANOVA was performed with a post hoc Tukey's Honestly Significant Difference (HSD) test, which indicated that of the coefficients of variation from the “redelivery” measurements, radiographic film was the only device that was statistically different from the other dosimeters (p=0.0001). An ANOVA and post hoc Tukey's HSD test were similarly performed on the “total” variability (Fig. 3(b)). Two groups of devices were apparent, with two devices (ArcCHECK and MapCHECK with original gantry angles delivered) not being significantly different from either group (p=0.004), as illustrated in a Venn diagram (Fig. 4). Film was again the most variable device, but was not statistically different from every other technique, as was the case in “redelivery” reproducibility.

When assessing the reproducibility of the QA systems, there is a difference between devices in how much variability is contributed from the delivery/readout and from the setup. Based on Eq. (1), the relative importance of each is summarized in Table 2. Film is the only dosimeter with a higher delivery/readout contribution than the setup (59% vs. 41%). All other QA systems show much greater variability stemming from the setup, with the planned‐angle MapCHECK demonstrating the highest (97% from setup vs. 3% from delivery/readout). It is of interest to note that the same physical dosimeter setup was used for AP field‐by‐field, planned‐angle, and AP composite MapCHECK. However, the differences in the way the results were obtained and analyzed led to various degrees of sensitivity to setup and readout, delivery, and analysis. For example, the variability in the AP composite MapCHECK and MapCHECK with original gantry angles delivered shows a higher dependence on setup than did the AP field‐by‐field MapCHECK.

**Table 2:** Isolating the error caused by setup alone from the redelivery and total coefficients of variation (CV) averaged across all patient plans

	*”Redelivery” Measurement CV*	*Setup CV*	*”Total Delivery” Measurement CV*	*% Variation from Delivery/Read‐out*	*% Variation from Setup*
AP field‐by‐field MapCHECK	0.09%	0.12%	0.15%	37%	63%
Planned‐angle MapCHECK	0.24%	1.3%	1.4%	3%	97%
cc04 ion chamber	0.13%	0.33%	0.36%	13%	87%
Radiographic film	1.5%	1.27%	2.0%	59%	41%
MIC IC Avg.	0.25%	0.38%	0.46%	31%	69%
ArcCHECK	0.36%	0.94%	1.0%	13%	87%
AP composite MapCHECK	0.14%	0.44%	0.46%	9%	91%

**Figure 5:**
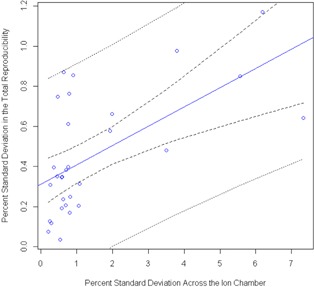
Regression performed to determine the ability to predict “total delivery” reproducibility, given a known percent standard deviation in dose across the multiple ion chamber (MIC) phantom ion chamber from the TPS. The inner pair of dotted lines is the 95% confidence interval in the linear fit, whereas the outer pair of lines is the 95% prediction confidence interval. This regression analysis failed to find a strong relationship between the standard deviation in dose across the ion chamber ROI and the reproducibility in the measurement; however, it is interesting to note that even at low‐dose gradients, there is the possibility for large variation in the IMRT QA measurement.

**Figure 6:**
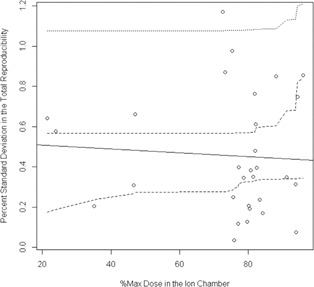
Regression analysis from the multiple ion chamber (MIC) phantom ion chamber measurements exploring whether knowing the percent of the plan's maximum dose in the chamber ROI can predict the “total delivery” reproducibility in the measurement. As with the standard deviation in the dose study, the inner pair of dotted lines is the confidence interval in the linear fit, whereas the outer pair of lines is the prediction confidence interval. This analysis also failed to show a strong relationship between placing an ion chamber in a high‐dose region and the reproducibility in the measurement.

Because a large number of ion chamber measurements were taken with the MIC phantom system, follow‐up evaluation was conducted on the relationship between the reliability of the measurement and the dose environment around that ion chamber (dose gradients and high‐dose regions). We compared the percent standard deviation in the “total” reproducibility measurements with the percent standard deviation in the dose across the ion chamber ROI (active volume) in the TPS. Using a linear model to fit the percent standard deviation in the measurement data to that found in the ROI, prediction confidence bands were calculated for the ability to predict a percent standard deviation in the measurement based on the percent standard deviation calculated across the ion chamber (Fig. 5). The R‐squared value for this model is only 0.36, showing very little relationship. This plot also shows that the prediction bands are wider than the plot in some places, revealing a lack of prediction power. No strong conclusions can be drawn from this result because the ion chamber points were not selected to test a wide range of dose gradients; the bulk of the data has a standard deviation of less than 1% as predicted by the TPS. However, this result is still of interest, and begs further study. Not only does there not appear to be much of a trend, but also even for measurements points in negligible dose gradients, the reproducibility in the measurement could sometimes be high (~1%). The analysis was done again with expansions of the ROI ion chamber volume by 1, 2, 3, and 5 mm to attempt to capture any effects from the local dose environment. There was no difference in the result for the 1, 2, 3, and 5 mm expansions. An additional examination of the ion chamber data also failed to show a relationship between “total delivery” reproducibility and the percent of the plan maximum dose in the ion chamber ROI (Fig. 6). This regression was not shown to be statistically significant (p=0.74), revealing a lack of linear fit in the data. Looking at the data points themselves, it is worth noting that when the ion chamber is placed at a region with a high percentage of the maximum dose (e.g., 96% of the plan's maximum dose), there is a possibility of seeing relatively large variability in the dose readings (e.g., 0.86%).

## IV. DISCUSSION

We were able to compare the potential for variation in QA results by performing a reproducibility study of various types of patient‐specific IMRT QA equipment and methods. The most salient finding was the greater variability in EDR2 film, which was significantly different from that in the other methods in both “total delivery” (a coefficient of variation of 2.0%) and “redelivery” (a coefficient of variation of 1.5%) studies. This is most likely influenced by the high degree of human interaction involved in the readout and analysis of film. The user has input on aligning the film to the calculated dose plane, selecting an ROI for the gamma analysis, and choosing a dose normalization point. An additional aspect inherent to film is the variation introduced from the film processing and the timing of the film development.^(7)^ No other QA method in this publication's study allowed for as much user latitude.

There were two types of reproducibility measured: “redelivery” and “total delivery.” After extracting the setup reproducibility from the “total delivery,” it was seen that the setup was more responsible for variability than the delivery/readout. The only exception to this was radiographic film, with its more involved readout. The “total delivery” study most closely mimics what a clinic could expect, since the equipment is set up from day to day to perform QA. The “redelivery” study represents the type of variation that would be seen if nothing was moved and only the plan was redelivered.

Apart from film, none of the other methods were significantly different in their coefficients of variation for the “redelivery” reproducibility. All methods, except film, have immediate readout capabilities, minimizing the degree of human interaction. However, this homogeneity decreased for the “total delivery” reproducibility, as the devices were divided into two significant groups, with two methods belonging to both groups (Fig. 4). All of the methods in Group A (Fig. 4) consist of a rotational delivery to a planar dosimeter, suggesting that the planar measurements with original gantry angles appear to be more sensitive to setup errors than when the fields are delivered perpendicularly. This may be due to the fact that there is an increase in the degrees of freedom in the motion of the dosimeter relative to the delivery of the beam. All point measurements and AP planar measurements belong in Group B with lower coefficients of variation.

In the clinic, when a plan gives suspicious QA results, sometimes it is redelivered with the assumption that there was an error in the initial delivery. This may or may not involve reset up of the device. This study shows the type of variation that could be expected from simply remeasuring the plan again. For example, if radiographic film is used as a dosimeter and is set up again for another measurement, a 2% change in the percent of pixels passing would be expected. Other devices showed less variation, but there is nevertheless some variability inherent in these measurements. The array devices typically had less than 1% variability on average (Fig. 3(b)), thus including setup uncertainties, repeated measurements should provide consistent percent of pixels passing within ~1%.

## V. CONCLUSIONS

By analyzing the coefficients of variation across seven different methods of patient‐specific IMRT QA, the goal of this research was to provide an intercomparison of the reproducibility of QA measurements. In terms of reproducibility, all methods demonstrated average coefficients of variation of less than 2% for the “total delivery” reproducibility. If a clinic performs a repeated measurement with a QA device, the percent of pixels passing could show up to a 2% variation on average, depending on the dosimeter. Film performed the poorest (2% total), while many planar devices showed total reproducibility much less than 0.5%. With the exception of film, the setup contributed a greater amount of variability than the delivery of the plan and readout of the dosimeter, often by a very large margin. The results from this study can provide clinics with greater insight into the expected reproducibility of their patient‐specific IMRT QA with respect to the dosimeter and method used.

## ACKNOWLEDGMENTS

I would like to thank the physics assistants (Scott Laneave, Nick Murray, Andrea Ohrt, and Luke Whittlesey) at MD Anderson for their generous help with this project. This work was supported by Public Health Service grants CA010953, CA081647, and CA21661 awarded by the National Cancer Institute, United States Department of Health and Human Services. Also this research is supported in part by the National Institutes of Health through MD Anderson's Cancer Center Support Grant NCI P30 CA016672.
